# Sociodemographic disparities and hospice referral practice patterns among patients with recurrent cervical cancer

**DOI:** 10.1016/j.gore.2026.102151

**Published:** 2026-06-19

**Authors:** Brittany H. File, Marianne R. Rara, Rakesh S. Nemmani, Jenny Chang, Robert E. Bristow

**Affiliations:** a Department of Obstetrics and Gynecology, University of California, Irvine Medical Center, Orange, CA, USA; b Department of Medical Education, University of California, Irvine College of Medicine, Irvine, CA, USA.; c Department of Medicine, School of Medicine, University of California Irvine, Orange, CA, USA; d Department of Obstetrics and Gynecology, Division of Gynecologic Oncology, University of California, Irvine Medical Center, Orange, CA, USA

**Keywords:** Cervical cancer, Hospice referral, Disparities, Sociodemographic, Racial and ethnic

## 1 Introduction

Cervical carcinoma (CC) is the fourth most common cancer affecting women worldwide (American Cancer Society, 2023). Although pap smears and human papillomavirus (HPV) vaccinations promote prevention and early disease detection, cervical cancer continues to persist with consequent poor outcomes. Approximately 6% of women with cervical cancer are initially diagnosed with primary metastatic disease, and approximately 33% of women with locally advanced disease will recur despite treatment with chemoradiation followed by brachytherapy (Gennigens et al., 2022; Li et al., 2022; Uppal et al., 2020). Metastatic/recurrent disease continues to be a significant cause of mortality, with a 5-year overall survival rate of only 17% (Gennigens et al., 2022).

Medical literature has suggested that social determinants of health including race and ethnicity, socioeconomic status, education level, and residential zip code strongly influence cervical cancer outcomes (Zreik et al., 2023; Yu et al., 2019). Despite these statistics, studies have demonstrated that there is a tendency toward later referral to palliative care for Black, Hispanic, and other non-White racial and ethnic groups, which can address adverse symptoms experienced during treatment improving quality of life, satisfaction with cancer care, and fewer unnecessary invasive measuring at end of life (Johnson, 2013; Islam et al., 2021). For minority patients referred to palliative care, they have been found to have more hospitalizations for cancer related symptoms suggesting suboptimal utilization of palliative care services (Islam et al., 2021). Maximizing quality of life through palliative and hospice utilization is an essential component of comprehensive care for patients with recurrent CC.

Investigating racial disparities in hospice care utilization, as well as their causes, provides further direction toward promoting more equitable end-of-life care. The objective of the current study was to investigate the impact of race, ethnicity, insurance-status, and age on the rate of hospice referrals among patients with recurrent cervical cancer.

## 2 Methods

Approval was obtained from the Institutional Review Board. Honest Broker queried electronic medical records (EMR) for patients with recurrent cervical cancer seen at The University of California, Irvine Health (UCI Health) from January 2000 to April 2024. An Honest Broker is an independent data intermediary that extracts de-identified or limited EMR data to ensure regulatory and privacy compliance. Because automated queries are limited by a reliance on structured ICD coding that can risk under- or over-inclusion, we subsequently conducted a meticulous manual chart review of the compiled list to clinically confirm all cervical cancer recurrences (Hassett et al., 2014; Dhir et al., 2008). The following characteristics were collected and evaluated: age at initial and recurrence diagnosis, self-reported racial and ethnic identification, insurance type, initial disease stage and pathology, adherence with treatment prior to recurrence, and treatment course including the length of time between recurrence diagnosis and hospice referral. Age in years was categorized into common age cutoffs <40, 40–49, 50–64, and 65+ to improve clinical interpretability and assess potential non-linear associations. Insurance coverage was merged into four groups: no insurance, Medicare, Medi-Cal, and private. Patient identified race and ethnicity were classified as Non-Hispanic White, Non-Hispanic Black, Hispanic, Asian, and self-identified Other. Pathology diagnosis rendered by UCI pathology at initial cervical cancer diagnosis, which was not re-reviewed for the purpose of this study, was defined as adenocarcinoma (AC), squamous cell carcinoma (SCC), or other/unknown. Staging at initial diagnosis was grouped into stage I or II, stage III or IV, and unknown.

Bivariate analyses of patients' characteristics by hospice referral status were conducted using the chi-square test or Fisher's exact test, as appropriate. Multivariable logistic regression models were fitted to evaluate the association between hospice referral and patients' characteristics after adjusting for all factors collected during chart review. Results are presented as adjusted odds ratios (aORs) with 95% confidence intervals (CIs).

Time-to-event analysis was conducted using multivariable Cox proportional hazards regression to evaluate factors associated with time from recurrence diagnosis to hospice referral. Time was calculated from the date of recurrent diagnosis to the date of hospice referral. Date of recurrent diagnosis was defined as the date of biopsy confirming recurrence or if no biopsy was performed, then the date of imaging-confirmed recurrence was used. Patients who did not experience hospice referral were censored at death or at the end of follow-up (June 13, 2024), whichever occurred first. Results are presented as adjusted hazard ratios (aHRs) with 95% confidence intervals (CIs). Failure curves were generated to compare the cumulative incidence of hospice referral over time across race/ethnicity subgroups.

All statistical analyses were performed using SAS version 9.4 (SAS Institute Inc., Cary, NC, USA). Statistical significance was defined as a two-sided *P* value <0.05.

## 3 Results

The initial Honest Broker query of EMR identified 307 patients who were seen at UCI Health from January 2000 to April 2024. Cases lacking definitive documentation of recurrent cervical cancer were excluded.

### 3.1 Demographics and disease characteristics

The final study cohort comprised 138 patients with recurrent cervical cancer, of whom 54 (38.1%) received a hospice referral and 84 (60.9%) did not (Table 1). The age distributions were balanced, and neither age at initial diagnosis nor age at recurrence significantly differed between patients who received a hospice referral and those who did not. Clinical and structural characteristics were also statistically similar across both groups. The majority of the cohort had Medi-Cal insurance, presented with Stage I or II disease at initial diagnosis, and exhibited squamous cell carcinoma pathology. There were no statistically significant differences between the referral and no-referral groups regarding insurance type, interpreter need, initial staging, or tumor pathology. Compliance with initial treatment was high overall (88.4%) and did not significantly differ based on final hospice referral status. Notably, race and ethnicity were the sole baseline demographic characteristic significantly associated with hospice referral (*p* = 0.0374). Among patients who did not receive a referral, Non-Hispanic White patients represented a large proportion (33.3%), whereas they accounted for only 14.8% of the cohort that did receive a referral. Conversely, Hispanic patients comprised exactly half (50%) of the cohort that received a hospice referral, compared to the 35.7% of the non-referral group.

**Table 1.** Bivariate analysis of demographic variables and hospice referral.

	Total (*n* = 138)		No referral (*n* = 84)		Had referral (*n* = 54)		
	N	%	N	Row % (n/84)	N	Row % (n/54)	P value⁎
Age at initial diagnosis							0.9713
<40	47	34.1%	29	34.5%	18	33.3%	
40–49	38	27.5%	24	28.6%	14	25.9%	
50–64	39	28.3%	23	27.4%	16	29.6%	
65+	14	10.1%	8	9.5%	6	11.1%	

Age at recurrence							0.919
<40	39	28.3%	24	28.6%	15	27.8%	
40–49	32	23.2%	19	22.6%	13	24.1%	
50–64	47	34.1%	30	35.7%	17	31.5%	
65+	20	14.5%	11	13.1%	9	16.7%	

Insurance							0.3922
No insurance	3	2.2%	3	3.6%	0	0.0%	
Medicare	29	21.0%	17	20.2%	12	22.2%	
Medi-Cal	66	47.8%	37	44.0%	29	53.7%	
Private	40	29.0%	27	32.1%	13	24.1%	

Race/Ethnicity							0.0374
Non-Hispanic White	36	26.1%	28	33.3%	8	14.8%	
Non-Hispanic Black	1	0.7%	0	0.0%	1	1.9%	
Hispanic	57	41.3%	30	35.7%	27	50.0%	
Asian	38	27.5%	21	25.0%	17	31.5%	
Others	6	4.3%	5	6.0%	1	1.9%	

Need interpreter							0.8746
No	88	63.8%	54	64.3%	34	63.0%	
Yes	50	36.2%	30	35.7%	20	37.0%	

Stage							0.1554
I or II	74	53.6%	47	56.0%	27	50.0%	
III or IV	59	42.8%	36	42.9%	23	42.6%	
Unknown	5	3.6%	1	1.2%	4	7.4%	

Pathology							0.618
AC	33	23.9%	18	21.4%	15	27.8%	
SCC	73	52.9%	47	56.0%	26	48.1%	
Others or unknown	32	23.2%	19	22.6%	13	24.1%	

Compliance with initial treatment							0.3434
Yes	122	88.4%	76	90.5%	46	85.2%	
No	16	11.6%	8	9.5%	8	14.8%	
Others	32	23.2%	19	22.6%	13	24.1%	

⁎ *P*-value from Chi Square test or Fisher's Exact test.

### 3.2 Hospice referral practice patterns

#### 3.2.1 Frequency to referral

**Fig. 1:**
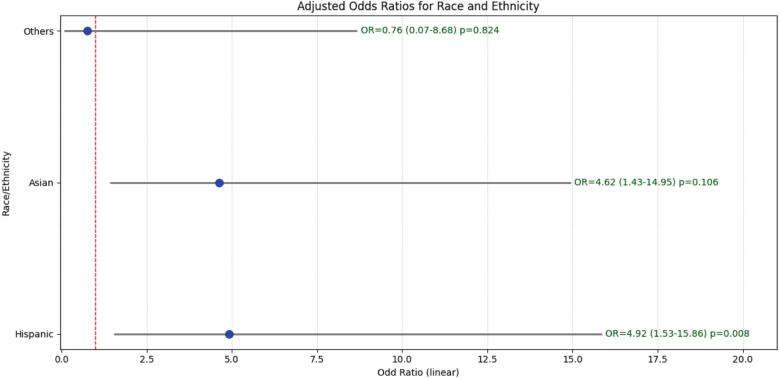
Adjusted odds ratios for race and ethnicity.

Multivariable logistic regression model revealed Hispanic and Asian patients have a 4.92 times (aOR, 95% (OR = 4.92, 95% CI = 1.53–15.86, *p* = 0.0076) and 4.62 times (aOR = 4.62, 95% CI = 1.43–14.95, *p* = 0.0106) higher odds of receiving a hospice referral compared to Non-Hispanic White counterparts after controlling for other demographic and clinical factors (Fig. 1). Logistic regression did not reveal significant associations between patient age at initial diagnosis, insurance coverage, initial tumor pathology, and initial staging when controlling for other demographic covariates (Table 2).

**Table 2.** Adjusted odds ratios for demographic variables predicting having hospice referral.

	Adjusted O.R.	95%. C.I.		p-value
Age at recurrence				
<40	Referent			
40–49	1.35	0.44	4.12	0.6032
50–64	0.94	0.33	2.63	0.8985
65+	1.14	0.27	4.82	0.8626

Insurance type				
No insurance	–	–	–	
Medicare	1.35	0.36	5.05	0.661
Medi-Cal	1.60	0.58	4.43	0.369
Private	Referent			

Race/Ethnicity				
Non-Hispanic White	Referent			
Non-Hispanic Black	–	–	–	
Hispanic	4.92	1.53	15.86	0.0076
Asian	4.62	1.43	14.95	0.0106
Others	0.76	0.07	8.68	0.8242

Need interpreter				
No	Referent			
Yes	0.48	0.18	1.26	0.137

Stage				
I or II	Referent			
III or IV	1.02	0.46	2.26	0.9715
Unknown	–	–	–	

Pathology				
AC	Referent			
SCC	0.48	0.18	1.30	0.151
Others or unknown	0.65	0.21	2.05	0.4614

#### 3.2.2 Temporal analysis

**Fig. 2:**
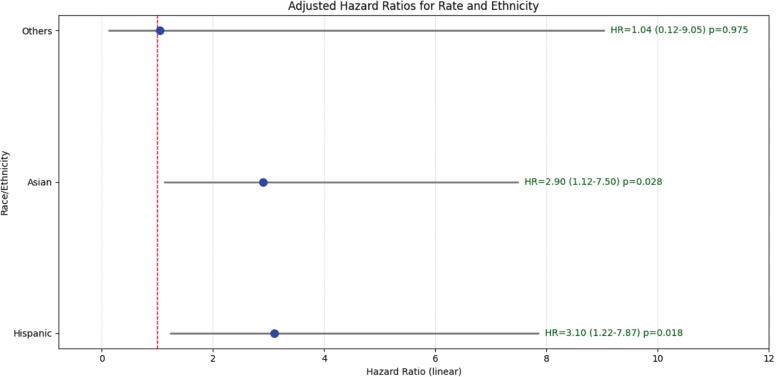
Adjusted hazard ratios for race and ethnicity.

**Fig. 3:**
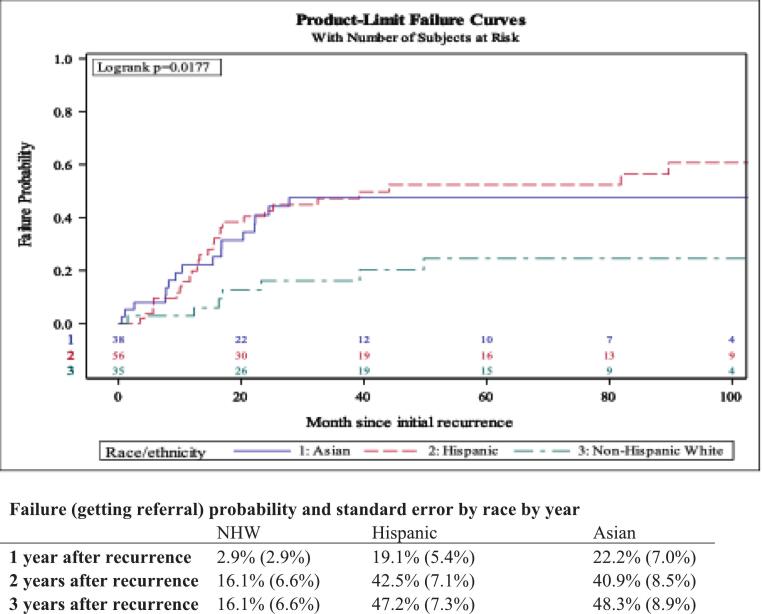
Failure curve analysis at two, three, and four years after recurrent diagnosis.

Time-to-referral analysis revealed Hispanic and Asian patients had 3.10 times (aHR = 3.10, 95% CI = 1.22–7.87, *p* = 0.0175) and 2.90 times (aHR = 2.90, 95% CI = 1.12–7.50, *p* = 0.0278) higher hazard of hospice referral than their White counterparts, respectively indicating earlier referral among Hispanic and Asian compared to White patients (Fig. 2). Time-to-referral analysis did not reveal significant associations between patient age at recurrent diagnosis, insurance coverage, initial tumor pathology, and initial staging when controlling for other demographic covariates (Table 3). Failure curve analysis demonstrated that the median time from cancer recurrence to hospice referral was approximately 16 months for non-Hispanic White patients compared to 14 months for Hispanic patients, representing a 2-month earlier referral among Hispanic patients. By 3 years after recurrence, 47% of Hispanic patients and 48% of Asian patients had received a hospice referral, compared to only 16% of non-Hispanic White patients. (Fig. 3).

**Table 3:** Adjusted hazard ratios for demographic factors associated with time from recurrence to referral, with censoring at last follow-up.

	Adjusted H.R.	95%. C.I.		p-value
Age at recurrence				
<40	Referent			
40–49	1.08	0.47	2.51	0.8533
50–64	0.91	0.40	2.05	0.8148
65+	1.41	0.52	3.84	0.4986

Insurance type				
No insurance	–	–	–	
Medicare	0.99	0.37	2.63	0.9801
Medi-Cal	1.53	0.71	3.27	0.2774
Private	Referent			

Race/Ethnicity				
Non-Hispanic White	Referent			
Non-Hispanic Black	–	–	–	
Hispanic	3.10	1.22	7.87	0.0175
Asian	2.90	1.12	7.50	0.0278
Others	1.04	0.12	9.05	0.9751

Need interpreter				
No	Referent			
Yes	0.74	0.36	1.51	0.4029
Stage				
I or II	Referent			
III or IV	1.15	0.62	2.13	0.6590
Unknown	2.13	0.59	7.75	0.2499

Pathology				
AC	Referent			
SCC	0.61	0.31	1.22	0.1595
Others or unknown	0.55	0.23	1.28	0.1645

## 4 Discussion

In this retrospective cohort study of patients with recurrent cervical cancer, we critically evaluated the relationship between demographic characteristics and end-of-life care utilization. Our primary finding demonstrates that Hispanic and Asian patients were referred to hospice significantly more frequently and earlier compared to their Non-Hispanic White counterparts.

Interestingly, this direction of disparity differs from broader body of existing end-of-life literature, which has documented a suboptimal hospice utilization and delayed enrollment among racial and ethnic minority groups. By establishing this unique utilization pattern within our cohort, this study highlights the distinct regional or institutional dynamics that can influence end-of-life care pathways in challenging oncologic disease. At this institution, where patients often have access to multidisciplinary care—including palliative care and social work—this may highlight the value of culturally informed care in helping patients utilize existing resources and navigate these complex end-of-life decisions. This is one of only a few studies that has investigated this topic.

Patients enrolled in hospice care demonstrate improved quality of life the longer they are enrolled (Wright et al., 2008). Despite the documented benefits of end of life (EOL) care utilization, previous work has shown that the majority of dying patients do not recall having EOL discussions, with even fewer noted at major academic centers (Wright et al., 2008). Palliative care use is especially low among patients with advanced and metastatic cervical cancer, with one prior study demonstrating only 11.6% utilization (Islam et al., 2021). Previous studies have also shown that palliative referrals reduce the incidences of emergency department visits, intensive care unit admissions, and inpatient stays for cervical cancer patients. Patients were also less likely to pass away in an acute setting (Bercow et al., 2021). These studies reinforce that referral to palliative care has shown beneficial outcomes, whether for comfort or reduction in hospital stays. Despite this, prior analysis has revealed discrepancies in hospice utilization, noting that racial minorities are less likely to enroll.(Taylor et al., 2016)

Many studies have shown that low-income women and Black and Hispanic women have higher incidence and mortality rates, are often diagnosed with later stage cervical cancer, and are more likely to die from the disease than White women (Yu et al., 2019; Shi et al., 2009; Matz et al., 2021; Beavis et al., 2017). The death rates for Black women and Native American women with cervical cancer are about 65% higher than for White women (McCarthy et al., 2010). Despite these statistics, studies have demonstrated that there is a tendency toward later referral to palliative care for minority patients with gynecologic cancer, which can address adverse symptoms experienced during treatment improving quality of life, satisfaction with cancer care, and fewer unnecessary invasive measuring at end of life (Islam et al., 2021; Flood and Rivard, 2018).

Additionally, studies have found that minority patients with gynecologic cancer referred to palliative care have more hospitalizations for cancer related symptoms, suggesting suboptimal utilization of palliative care services. One prior study demonstrated that minority gynecologic cancer patients are less likely to utilize hospice services at the end-of-life likely due to various factors including cultural values, knowledge barriers, economic factors, and mistrust of the health care system (Washington et al., 2008).

Available evidence investigating end-of-life care in Hispanic and Asian communities are both limited and mixed, particularly in the space of gynecologic oncology. Cohen et al. completed a systematic review on racial and ethnic disparities in hospice care (all conditions) and identified disparities in 12 of the 13 studies with African American patients utilizing hospice services less than White patients, though lacked power to draw conclusions (Cohen, 2008). Additional studies specifically investigating hospice care in all cancer have found similar disparities with racial and ethnic minorities less likely to be enrolled and utilize hospice services (Wang et al., 2019; Ngo-Metzger et al., 2003; Dinicu et al., 2023; Robison et al., 2023). Smith et al. found Black and Hispanic patients with all advanced cancer were less likely to have an advanced care plan and more likely to desire life-prolonging care compared to White patients (Smith et al., 2008). When evaluating the more robust literature related to palliative care utilization among patients with gynecologic cancers the evidence is mixed; however, trends toward evidence detailing inequity (Islam et al., 2021; Taylor et al., 2016; Flood and Rivard, 2018; Lefkowits et al., 2014; Rosenfeld et al., 2018).

Islam et al. examined palliative care utilization among 124,729 patients with metastatic gynecologic cancers using National Cancer Database (NCDP) and found that Hispanic patients with metastatic cervical cancer were significantly less likely to receive palliative care compared to Non-Hispanic White patients. In contrast, our analysis demonstrated that Hispanic patients with recurrent cervical cancer were more likely to receive a hospice referral compared to Non-Hispanic White patients. While these findings appear discordant, several important differences between the studies may explain the discrepancy.

Islam et al. assessed receipt of palliative care, whereas our study examined hospice referral. Although related, referral to palliative care and referral to hospice occur at different points along the continuum of care and may be influenced by different patient, provider, and system-level factors. Islam et al. additionally utilized a national database encompassing substantial geographic and institutional variation in palliative care access and referral practices across the United States, whereas our study was conducted within a single academic health system in Southern California. Regional differences in patient demographics, healthcare infrastructure, and availability of supportive care resources may influence referral patterns and contribute to variation in observed racial and ethnic disparities.

Our findings are more consistent with those of Tabuyo-Martin et al. which conducted a retrospective cohort study at a tertiary referral center examining palliative medicine referral among patients with advanced or recurrent gynecologic malignancies (Tabuyo-Martin et al., 2023). Although race and primary language were not associated with referral to palliative medicine, the authors observed a higher proportion of hospice referrals among Black patients relative to White patients. Taken together, these findings suggest racial and ethnic disparities observed in large national datasets may not uniformly manifest at the institutional or regional level. Broader literature has demonstrated substantial geographic variation in end-of-life care and hospice utilization, suggesting that regional factors and healthcare system may play a major role in shaping patterns of care (Connor et al., 2007; Wang et al., 2015; Stiefel et al., 2008). This supports the possibility that multidisciplinary care models and palliative care pathways may mitigate disparities in hospice referral among patients.

We observed that Hispanic and Asian patients were approximately five times more likely to have received a hospice referral when compared to Non-Hispanic White patients when controlling for variables including insurance status, initial staging and pathology, and adherence with initial treatment plan. We similarly found that Hispanic and Asian patients were receiving their hospice referral approximately three times faster than Non-Hispanic White patients.

Our findings raise the question of whether higher hospice referral rates among Hispanic and Asian patients reflect differing end-of-life goals shaped by cultural values, or physician-driven variation in referral practices. Elting et al. examined hospice utilization among patients with cancer (non-gynecologic) enrolled in Medicare and demonstrated that racial and ethnic disparities persisted even after accounting for factors including age at diagnosis, sex, year of death, cancer type, disease stage, survival duration, and dual insurance eligibility (Elting et al., 2020). These results suggest that hospice enrollment is influenced not only by clinical characteristics but also by personal preferences, cultural beliefs, societal norms, and the dynamics of physician–patient relationships. Within our academic institution—where many patients are concurrently followed by palliative care specialists and social workers—our findings may underscore the importance of culturally informed, multidisciplinary relationships in helping patients navigate potential barriers to hospice enrollment.

A key limitation of this study is its retrospective design and the absence of granular clinical metrics, including progress-free survival, overall survival, enrollment in clinical trials, and the exact number of total lines of salvage therapy received after recurrence. Consequently, a shorter or longer interval from recurrence to hospice referral cannot be definitively labeled as a measure of clinical appropriateness, as these timelines are heavily influenced by extended investigational protocols or later-line therapeutic choices. If survival outcomes are equivalent at our institution, earlier hospice referral among racial and ethnic minority patients may reflect timely and effective linkage to supportive resources that enhance end-of-life care. Our dataset also does not capture other critical healthcare utilization metrics established in the end-of-life literature, such as the timing of outpatient palliative care referrals, inpatient palliative consultations, emergency department visits, or ICU admissions near the end of life. Because hospice enrollment alone provides an incomplete picture of total end-of-life care, evaluating these adjacent services represents an essential area for future multi-center research to fully map how demographics intersect with comprehensive healthcare utilization. Furthermore, as a single-institution chart review our findings may reflect unique regional utilization patterns and are constrained by a modest sample size. This limited our ability to robustly evaluate certain subsets of patients, such as Non-Hispanic Black patients, who represented a very small proportion of our cohort. A larger, multi-institutional evaluation expanding to all gynecologic malignancies is needed to fortify these results. Additionally, the small sample size within certain demographic subgroups resulted in wide confidence intervals for some measures of association, which limits the statistical precision of those specific estimates and requires cautious interpretation.

A distinct strength of this localized chart review over a much larger de-identified national database is that it granted direct access to a current, unique data set with a wide range of socioeconomic variables that we could evaluate including insurance status and interpreter usage. Ultimately, additional prospective research is required to clarify the precise patient- and provider-specific characteristics that drive these utilization disparities and to determine how to best optimize equitable end-of-life outcomes.

## Study approval statement

This study protocol was reviewed and approved by the University of California Institutional Review Board.

## Consent to publish statement

Written informed consent was obtained from the patient for publication of this case report. A copy of the written consent is available for review by the Editor-in-Chief of this journal on request.

## CRediT authorship contribution statement

**Brittany H. File:** Writing – review & editing, Writing – original draft, Supervision, Methodology, Investigation, Formal analysis, Data curation, Conceptualization. **Marianne R. Rara:** Writing – original draft, Formal analysis, Data curation. **Rakesh S. Nemmani:** Writing – original draft, Formal analysis, Data curation. **Jenny Chang:** Software, Methodology, Formal analysis. **Robert E. Bristow:** Writing – review & editing, Supervision, Conceptualization.

## Funding sources

The study was not supported by any sponsor or funder.

## Declaration of competing interest

The authors declare that they have no known competing financial interests or personal relationships that could have appeared to influence the work reported in this paper.

## Data Availability

Data sharing is not applicable to this article as no datasets were generated or analyzed during the current study.
